# The circadian regulator *Bmal1* in joint mesenchymal cells regulates both joint development and inflammatory arthritis

**DOI:** 10.1186/s13075-018-1770-1

**Published:** 2019-01-06

**Authors:** Laura E. Hand, Suzanna H. Dickson, Anthony J. Freemont, David W. Ray, Julie E. Gibbs

**Affiliations:** 1 Division of Diabetes, Endocrinology and Gastroenterology, School of Medical Sciences, Faculty of Biology, Medicine and Health, University of Manchester, AV Hill Building, Oxford Road, Manchester, UK; 2 Division of Cell Matrix Biology and Regenerative Medicine, School of Biological Sciences, Faculty of Biology, Medicine and Health, University of Manchester, Manchester, UK; 3 Oxford Centre for Diabetes, Endocrinology and Metabolism, University of Oxford, Oxford, OX3 7LE UK; 4 NIHR Oxford Biomedical Research Centre, John Radcliffe Hospital, Oxford, OX3 9DU UK; 5 The Lydia Becker Institute of Immunology and Inflammation, The University of Manchester, Manchester, UK

**Keywords:** BMAL1, Circadian, Fibroblast-like synoviocytes, Chondrocyte, Arthritis, Macrophage, Synovium

## Abstract

**Background:**

The circadian clock plays a crucial role in regulating physiology and is important for maintaining immune homeostasis and responses to inflammatory stimuli. Inflammatory arthritis often shows diurnal variation in disease symptoms and disease markers, and it is now established that cellular clocks regulate joint inflammation. The clock gene *Bmal1* is critical for maintenance of 24-h rhythms and plays a key role in regulating immune responses, as well as in aging-related processes. Fibroblast-like synoviocytes (FLS) are circadian rhythmic joint mesenchymal cells which are important for maintenance of joint health and play a crucial role in the development of inflammatory arthritis. The aim of this study was to investigate the importance of the joint mesenchymal cell circadian clock in health and disease.

**Methods:**

Mice were generated which lack *Bmal1* in Col6a1-expressing cells, targeting mesenchymal cells in the ankle joints. Joints of these animals were assessed by X-ray imaging, whole-mount staining and histology, and the composition of the synovium was assessed by flow cytometry. Arthritis was induced using collagen antibodies.

**Results:**

*Bmal1* deletion in joint mesenchymal cells rendered the FLS and articular cartilage cells arrhythmic. Targeted mice exhibited significant changes in the architecture of the joints, including chondroid metaplasia (suggesting a switch of connective tissue stem cells towards a chondroid phenotype), reductions in resident synovial macrophages and changes in the basal pro-inflammatory activity of FLS. Loss of *Bmal1* in FLS rendered these resident immune cells more pro-inflammatory in response to challenge, leading to increased paw swelling, localised infiltration of mononuclear cells and enhanced cytokine production in a model of arthritis.

**Conclusions:**

This study demonstrates the importance of *Bmal1* in joint mesenchymal cells in regulating FLS and chondrocyte development. Additionally, we have identified a role for this core clock component for restraining local responses to inflammation and highlight a role for the circadian clock in regulating inflammatory arthritis.

**Electronic supplementary material:**

The online version of this article (10.1186/s13075-018-1770-1) contains supplementary material, which is available to authorized users.

## Background

The circadian clock enables organisms to regulate their physiology in synchrony with the changing 24-h environment. This biological timer regulates a wide range of physiological processes, including metabolism and immunity [1, 2]. Multiple different cell types possess the clockwork machinery, a network of genes forming a transcriptional-translational feedback loop. In mammals the core clock genes include *Bmal1*, *Clock*, *Cryptochrome* (*Cry*) and *Period* (*Per*), where *Bmal1* is the only non-redundant gene. Mice lacking *Bmal1* from pre-natal development are behaviourally arrhythmic in the absence of an entraining light/dark cycle, and show loss of rhythmic physiology [3]. In addition to co-ordinating circadian rhythms, *Bmal1* regulates other physiological functions, and these global knockout mice have reduced lifespan and fertility and exhibit pathologies affecting the eyes, brain and bone [4, 5]. This includes a progressive non-inflammatory arthropathy resulting in joint ankylosis [5]. Interestingly, this phenotype persists if *Bmal1* is rescued in brain or muscle [6], but is absent if *Bmal1* is only deleted after birth [7].

In the present study, we explored the contribution of *Bmal1* in fibroblast-like synoviocytes (FLS) to joint architecture. FLS are found within the lining of the synovium, the thin organised membrane located between the joint cavity and joint capsule. They are stromal cells of mesenchymal origin, producing a range of extracellular matrix components and secreted factors essential to maintaining the normal environment of the synovial fluid and articular surface [8]. FLS play a critical role in the pathogenesis of inflammatory arthritis, producing inflammatory mediators which contribute to the recruitment and activation of leucocytes, cartilage breakdown and joint remodelling [9]. It is well established that the core clock proteins (PERIOD1/2, BMAL1 and CLOCK) are expressed by FLS [10, 11], and we and others have shown that these immunoregulatory cells are circadian rhythmic [11–13]. Intriguingly, there is mounting evidence that under chronic inflammatory conditions, such as rheumatoid arthritis, these intrinsic timers are disrupted [10, 11, 13–15].

By deleting *Bmal1* in Col6a1-expressing cells we rendered joint mesenchymal cells (FLS and articular chondrocytes) arrhythmic. This targeted deletion had profound effects on joint architecture, homeostasis and inflammatory joint disease, highlighting the critical importance of the joint mesenchymal cell clock in health and disease.

## Methods

### Mice

B6.Cg-Tg(Col6a1-cre)1Gkl/Flmg mice, referred to hereinafter as Col6a1^cre/+^ mice, were purchased from the European Mutant Mouse Archive repository as frozen embryos and re-derived in-house. These mice, generated by Prof. G. Kollias [16], express Cre recombinase under the control of a collagen VI promoter cassette known to drive gene expression in mesenchymal cells in the ankle joints, mainly fibroblast-like cells but also articular chondrocytes [16, 17]. Bmal1^flox/flox^ PER2::luc mice (as described previously [18]) were bred with Col6a1^Cre/+^ mice to produce Bmal1^flox/flox^ PER2::luc Col6a1 Cre^+/−^ mice (Col6a1-Bmal1^−/−^) and Bmal1^flox/flox^ PER2::luc Col6a1 Cre^−/−^ mice (wild-type counterparts). Global *Bmal1*^−/−^ mice [19] were bred as heterozygous pairs to produce *Bmal1*^−/−^ and wild-type littermates. All animal procedures were carried out in accordance with the United Kingdom Animals (Scientific Procedures) Act 1986 and were subject to local ethical review by the University of Manchester Animals Welfare and Ethical Review Board. Mice were maintained under standard 12-h/12-h light/dark lighting with *ad libitum* access to standard chow. Unless stated otherwise, male mice aged 8–20 weeks were used.

### Murine FLS cultures

Mouse FLS were cultured as described previously [12]. In brief, mice were killed, and their skin was removed from the hind paws, which were then cut 1 mm above the ankle joint and placed into Hanks’ balanced salt solution. After a wash, the paws were dissected between the toe and ankle joints using a scalpel blade, and the dissected tissue was placed into DMEM (containing 1% penicillin-streptomycin and 10% FBS) with 10 mg/ml collagenase (from *Clostridium histolyticum* type IV; Sigma-Aldrich, Gillingham, UK) and placed into a shaking incubator for 1.5 h at 37 °C. Cells (and some tissue debris) were pelleted by centrifugation, re-suspended in DMEM and plated out. Cells were cultured in DMEM to passage 3 before further experimental use, in order to obtain a purified population of FLS.

### Bead sorting of FLS

Cultured mouse FLS (passage 3) were purified further by labelling them with a phycoerythrin (PE)-CD90.2 antibody (clone 30-H12) and positively selecting PE-labelled cells (EasySep PE Positive Selection Kit, as per kit instructions; STEMCELL Technologies, Vancouver, BC, Canada) for further culture. Bead-sorted FLS were used for Western blotting of the BMAL1 protein, as well as for qPCR analysis and in tumour necrosis factor (TNF)-α stimulation experiments (*see below*).

### Murine macrophages

Mice were killed, and the peritoneal cavity was lavaged with two washes of 5 ml of RPMI (containing 1% penicillin-streptomycin and 10% FBS). The recovered media was  centrifuged (432 × *g* for 10 min). For RNA analysis, the resultant pellet of peritoneal exudate cells was lysed immediately, and RNA extraction was carried out (as described below). For photomultiplier tube recordings, the pellet was re-suspended in RPMI, and cells were plated out into a 35-mm dish. After 2-h incubation at 37 °C, non-adherent cells were removed via three washes in warmed RPMI. For bone marrow-derived macrophages, mice were killed and the femur and tibia were removed. The bone marrow was flushed out with DMEM (containing 1% penicillin-streptomycin and 10% FBS), and the effluent was centrifuged (432 × *g* for 10 min). The pelleted cells were re-suspended in media containing 50 ng/ml macrophage colony-stimulating factor (M-CSF) (eBioscience, San Diego, CA, USA). The media were replaced after 3 days, and on day 6, the bone marrow-derived macrophages were scraped from the flask and lysed to extract protein for Western blot analysis (described below).

### Bioluminescence recordings

FLS (passage 3) and peritoneal macrophages were plated out on 35-mm dishes. Primary murine lung fibroblasts were cultured as described elsewhere [20] and plated out on 35-mm dishes. Femoral head tissue was placed directly onto tissue culture inserts within the dishes. Cells and tissues were synchronised (200 nM dexamethasone, 1 h), and the media were replaced with recording media containing luciferin [21]. Dishes were sealed over with a glass coverslip using vacuum grease [22]. Bioluminescence from FLS, femoral head tissue and lung fibroblasts was recorded every minute using photomultiplier tubes. Bioluminescence data from peritoneal macrophages were collected and analysed using a LumiCycle (ActiMetrics, Wilmette, IL, USA). Data were plotted using Prism software (GraphPad Software, La Jolla, CA, USA).

### Western blot analysis

Cultured FLS and bone marrow-derived macrophages were lysed using a radioimmunoprecipitation assay buffer (Sigma-Aldrich) containing cOmplete Mini, EDTA-free Protease Inhibitor Cocktail Tablets (Roche Diagnostics, Mannheim, Germany). Protein concentrations of cell lysates were measured with a bicinchoninic acid protein quantification kit (Merck, Kenilworth, NJ, USA), and an equal amount of protein was separated on SDS-polyacrylamide gels and transferred to nitrocellulose membranes (Thermo Fisher Scientific, Waltham, MA, USA). Membranes were incubated with primary antibody against BMAL1 (D2L7G; Cell Signaling Technology, Danvers, MA, USA) and β-ACTIN (ab8226; Abcam, Cambridge, UK), followed by horseradish peroxidase-conjugated secondary antibody, and imaged using enhanced chemiluminescence (Clarity; Bio-Rad Laboratories, Hercules, CA, USA).

### TNF-α stimulation of FLS

Bead-sorted FLS were stimulated with 10 ng/ml murine TNF-α (Sigma-Aldrich). Eight hours later the supernatant was removed. Cytokine levels in supernatants were quantified using the Bio-Plex Pro Mouse Cytokine 23-plex assay (Bio-Rad Laboratories) as per kit instructions, and run on a Bio-Plex 200 System. CXCL5 and M-CSF levels in supernatant were quantified separately using DuoSet enzyme-linked immunosorbent assay (ELISA) kits as per kit instructions (R&D Systems, Minneapolis, MN, USA).

### Collagen antibody-induced arthritis

Male mice (aged 10 weeks) were administered a cocktail of collagen antibodies (ArthritoMab; MD Biosciences, Egg, Switzerland) intravenously (4 mg/mouse) on day 0, and lipopolysaccharide (100 μg/mouse) intraperitoneally on day 3. Both treatments were administered during the mid-light phase (Zeitgeber time [ZT] 6). Each paw was scored for disease severity using a 4-point scale (1 = inflammation of one digit; 2 = inflammation of more than one digit; 3 = inflammation and swelling of the footpad with or without involvement of one or more digits; 4 = severe inflammation and swelling of the footpad and ankle with or without involvement of one or more digits). Each paw score was totalled to produce an animal score. Paw thickness was measured in the hind limbs using a thickness gauge. Mice were killed on day 7, when serum was harvested along with the left hind paw for RNA analysis and the right hind paw was harvested for flow cytometry. ELISAs for interleukin (IL)-6 in mouse serum were performed using DuoSet ELISAs (R&D Systems) as per kit instructions.

### qPCR

RNA was extracted from snap-frozen limbs and Peyer’s patches using TRIzol reagent (Life Technologies, Carlsbad, CA, USA). Frozen limbs were first pulverised using a pestle and mortar containing liquid nitrogen, and the resulting material was transferred to a Lysing Matrix D tube (MP Biomedicals, Santa Ana, CA, USA) and homogenised in TRIzol reagent using a FastPrep-24 machine (MP Biomedicals). Peyer’s patches were added directly to the Lysing Matrix D tubes containing TRIzol reagent for homogenisation. RNA was extracted using the standard TRIzol procedure. RNA was extracted from cultured primary cells using RNeasy kits (Qiagen, Hilden, Germany). After DNase treatment (DNase I, Thermo Fisher Scientific), RNA was converted to complementary DNA (cDNA) using the High-Capacity RNA-to-cDNA Kit (Thermo Fisher Scientific). qPCR was performed using a StepOne Real Time PCR system (Thermo Fisher Scientific) with TaqMan primers (*see* Additional file 1: Table S1). The housekeeping gene *Gapdh* was used to normalise data.

### Flow cytometry

Whole limbs were dissected at the joints, and the tissue was digested in collagenase (as described earlier). Isolated cells were counted using a nucleocounter NC-250 (ChemoMetec, Allerod, Denmark). A sample (5 μl) of the cell suspension was diluted in PBS (1:4), and a solution of acridine orange and 4′,6-diamidino-2-phenylindole (Solution 18; ChemoMetec) was added to provide counts of total and dead cells, respectively. For flow cytometric analysis, the remaining cells were subjected to live/dead staining (LIVE/DEAD Fixable Blue Dead Cell Stain Kit; Thermo Fisher Scientific) for 20 min. ArC Amine Reactive Compensation Beads (Thermo Fisher Scientific) were used to obtain compensation controls for live/dead staining. After washing off the live/dead stain, an Fc block (CD16/CD32) was applied for 15 min, and then cells were stained with a cocktail of antibodies (Additional file 1: Tables S2 and S3). Cells were analysed on a BD LSR II flow cytometer (BD Biosciences, San Jose, CA, USA) after compensation set-up with OneComp eBeads (Thermo Fisher Scientific). Gating strategies are outlined in Additional file 1: Figures S4 and S5. Data were analysed using FlowJo software (FlowJo, Ashland, OR, USA). In some experiments FLS (CD45^−^CD90.2^+^) were sorted from these samples (BD Influx cell sorter; BD Biosciences) into lysis buffer, and RNA was prepared using RNeasy Plus Micro kits (Qiagen).

### Histology

The skin was removed from hind limbs, which were then placed into formalin overnight. The next day, tissue was transferred to OSTEOSOFT (VWR, Radnor, PA, USA) for 2 weeks to allow de-calcification. De-calcified tissue was processed and embedded into paraffin wax. Sections (5 μm) were cut and mounted onto glass slides. After rehydration, slides were subjected to H&E staining or Safranin O staining using standard protocols. Slides were imaged on a Leica DM2000 microscope, and images were captured with a Leica DFC295 camera (Leica Microsystems, Wetzlar, Germany).

### X-ray imaging

Mice were killed and X-rayed on a Faxitron MX-20 (32 kV for 55 s) (Faxitron Bioptics, Tucson, AZ, USA). Scanned X-ray films were analysed using ImageJ software. Using these images, pixel intensity was quantified  in a standardised size ROI superior to the calcaneus or between vertebral disks. This value was adjusted for background intensity.

### Whole-mount staining

Whole-mount staining was performed as described elsewhere [23]. In brief, skin and adipose tissue were removed from the hind limbs, and limbs were detached at the pelvis. Limbs were fixed in 95% ethanol for 48 h, then placed into 100% acetone (2 days). They were stained with Alcian blue solution for 3 days (0.03% wt/vol Alcian blue, 80% ethanol, 20% glacial acetic acid). After a brief wash (70% ethanol) they were de-stained overnight in 95% ethanol. Limbs were then placed in potassium hydroxide (1% wt/vol) for 4 h before staining with Alizarin red solution (0.005% wt/vol Alizarin red in 1% wt/vol KOH) for 4–5 days. After a wash in distilled water they were placed in 1% KOH for 2 days to begin clearing. Clearing solution was changed weekly, with decreasing amounts of KOH and increasing amounts of glycerol, until after 3 weeks limbs were placed in 100% glycerol for imaging and storage.

### Statistical analysis

Flow cytometry data were analysed using FlowJo version 10 software. All other data were analysed using Prism 7 software. Data are presented as mean ± SEM. Where statistical tests were used, statistical significance is annotated as follows: *P < 0.05, **P < 0.01, ***P < 0.005.

## Results

### *Bmal1* deletion renders FLS arrhythmic

**Fig. 1:**
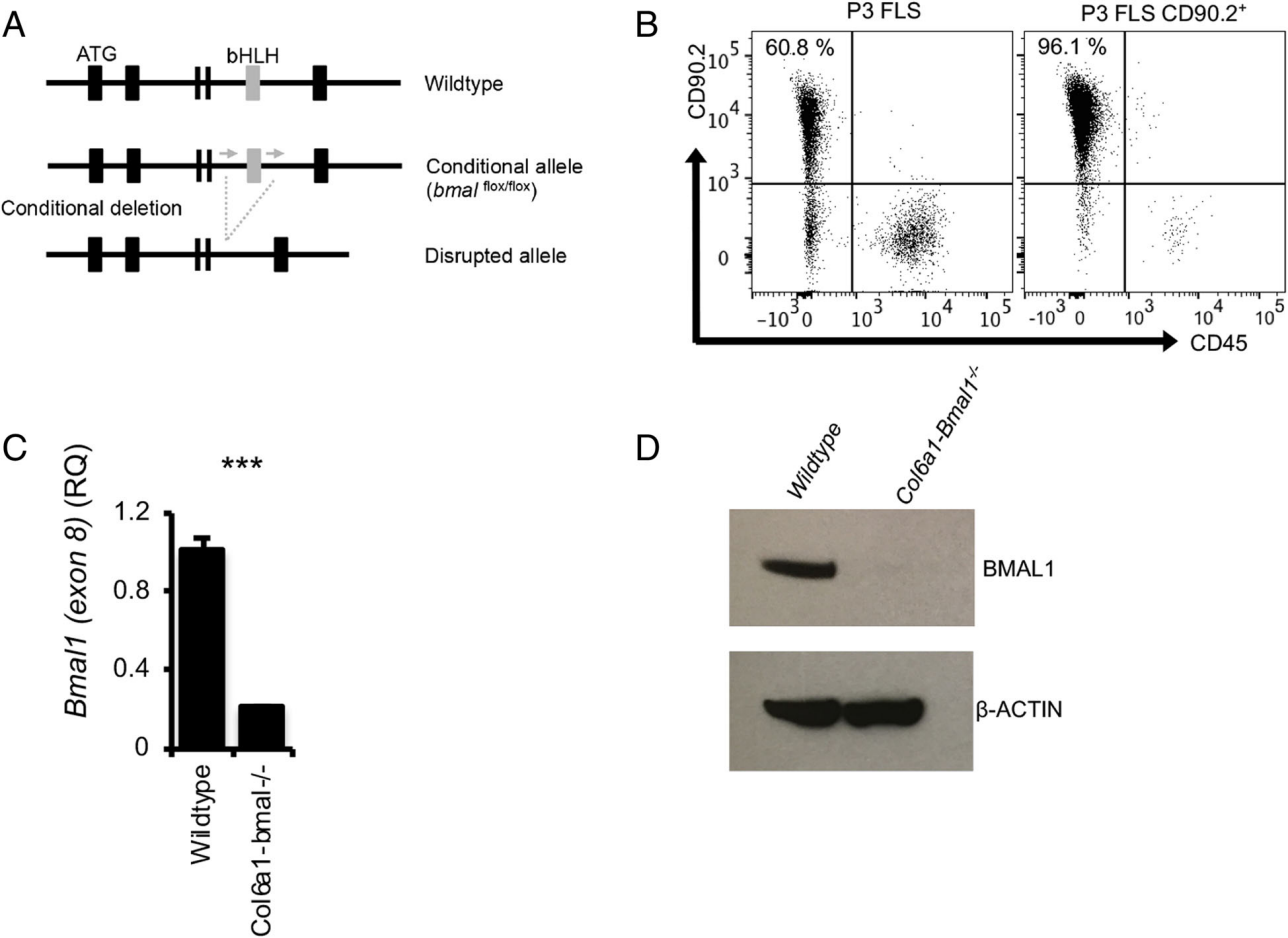
Targeting *Bmal1* for deletion in joint mesenchymal cells. **a** The basic helix-loop-helix–encoding exon of *Bmal1* was targeted for deletion in cells expressing *Col6a1*. **b** Fibroblast-like synoviocytes (FLS) were cultured from mouse hind limbs to passage 3, and assessed by flow cytometry for the absence of leukocytes (CD45^−^) and presence of the FLS marker CD90.2. CD90.2^+^ cells were then selected for further culture. **c** qPCR confirmed significant reduction in *Bmal1* transcript in targeted cells (*n* = 3; unpaired *t* test), which corresponded with (**d**) loss of BMAL1 protein

Mice were generated which lack the core clock component *Bmal1* in Col6a1-expressing cells (Fig. 1a). To confirm efficient gene targeting, FLS were cultured to passage 3 (61% purity) and were then further purified by selecting CD90.2^+^ cells (96% purity) (Fig. 1b). These cells were then subjected to qPCR and Western blot analysis. Results showed significantly reduced expression of the targeted gene (exon 8) (Fig. 1c) and reduced BMAL1 protein (Fig. 1d).

**Fig. 2:**
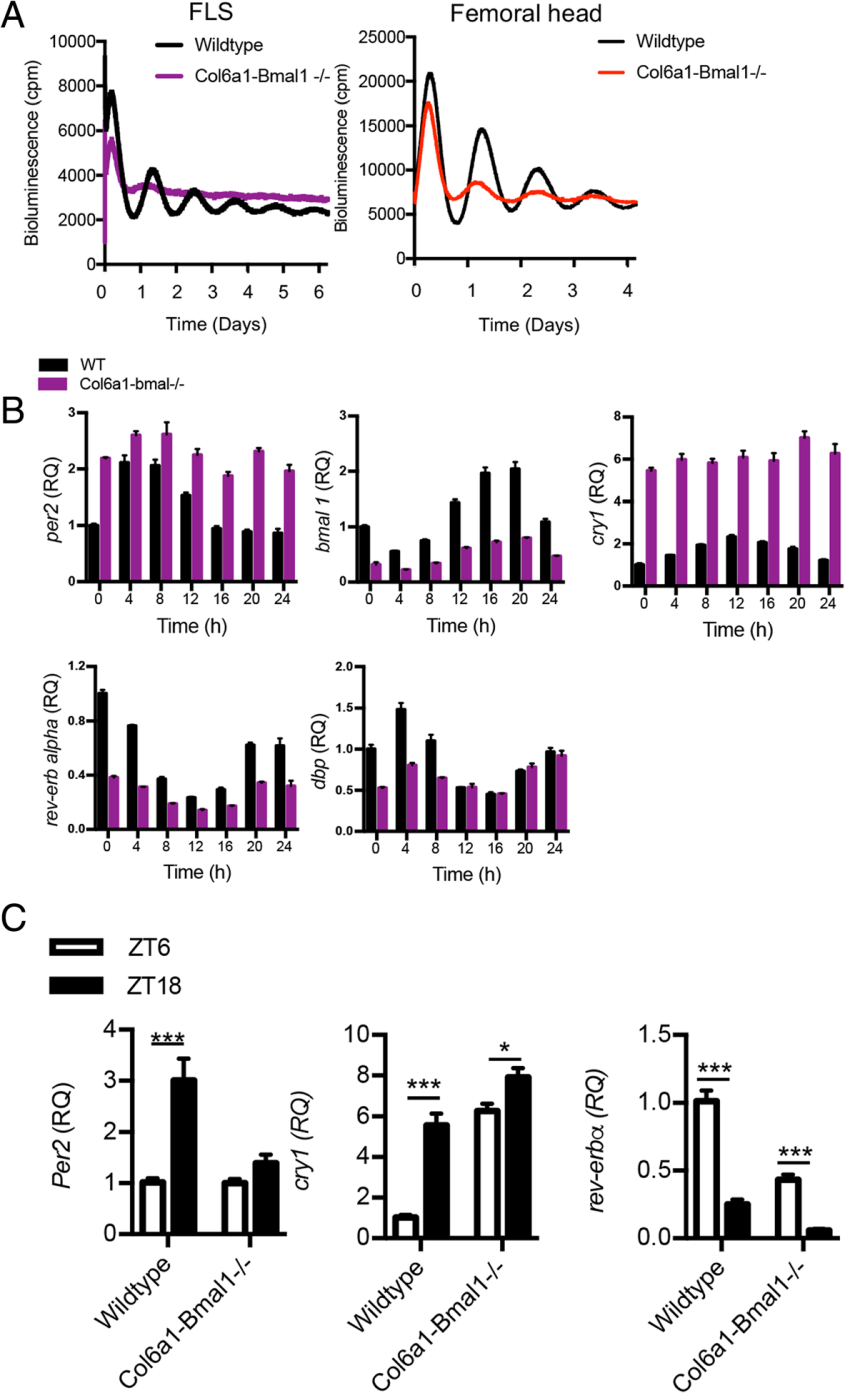
Deletion of *Bmal1* in joint mesenchymal cells renders fibroblast-like synoviocytes (FLS) and chondrocytes arrhythmic. **a** Cultured and purified FLS and femoral head tissue from Col6a1-*Bmal1*^−/−^ and wild-type animals (on a PER2::luc background) were placed under photomultiplier tubes to record bioluminescence (representative of *n* = 3/genotype). **b** Cultured FLS were harvested every 4 h for RNA extraction, and clock gene expression was profiled. Values are made relative to expression in wild-type cells at time point 0. **c** Clock gene expression in FLS (CD45^−^CD90.2^+^) sorted from the limbs of male and female mice (9–13 weeks) at ZT6 and ZT18 (*n* = 4–6). Two-way analysis of variance and post hoc Bonferroni correction were performed

Breeding these mice onto a PER2::luc background [21] permitted bioluminescence monitoring of cells and tissue from these mice as a readout of clock activity. Real-time luciferase recording under photomultiplier tubes confirmed *Bmal1* deletion rendered FLS arrhythmic (Fig. 2a). Conversely, lung fibroblasts remained rhythmic (Additional file 1: Figure S1a). The femoral head, containing chondrocytes, also showed dampened PER2::luc rhythms (Fig. 2a). In further studies, cultured and purified FLS (as described above) were sampled every 4 h. As expected, these showed reduction in *Bmal1* expression, loss of circadian oscillation in *Dbp* and *Rev-erbα*, and increased, non-oscillatory expression of *Cry1* and *Per2* (both repressed by BMAL1) (Fig. 2b). Finally, to confirm that the targeting rendered these cells arrhythmic in vivo, FLS were sorted by fluorescence-activated cell sorting (CD45^−^CD90.2^+^) from naïve limbs at opposing times of day (ZT6, mid-light versus ZT18, mid-dark). qPCR analysis revealed loss of rhythmic *Per2* and impaired rhythmicity of *Cry1* and *Rev-erbα* (Fig. 2c), confirming results from in vitro approaches.

### Loss of Bmal1 in FLS significantly affects joint structure

**Fig. 3:**
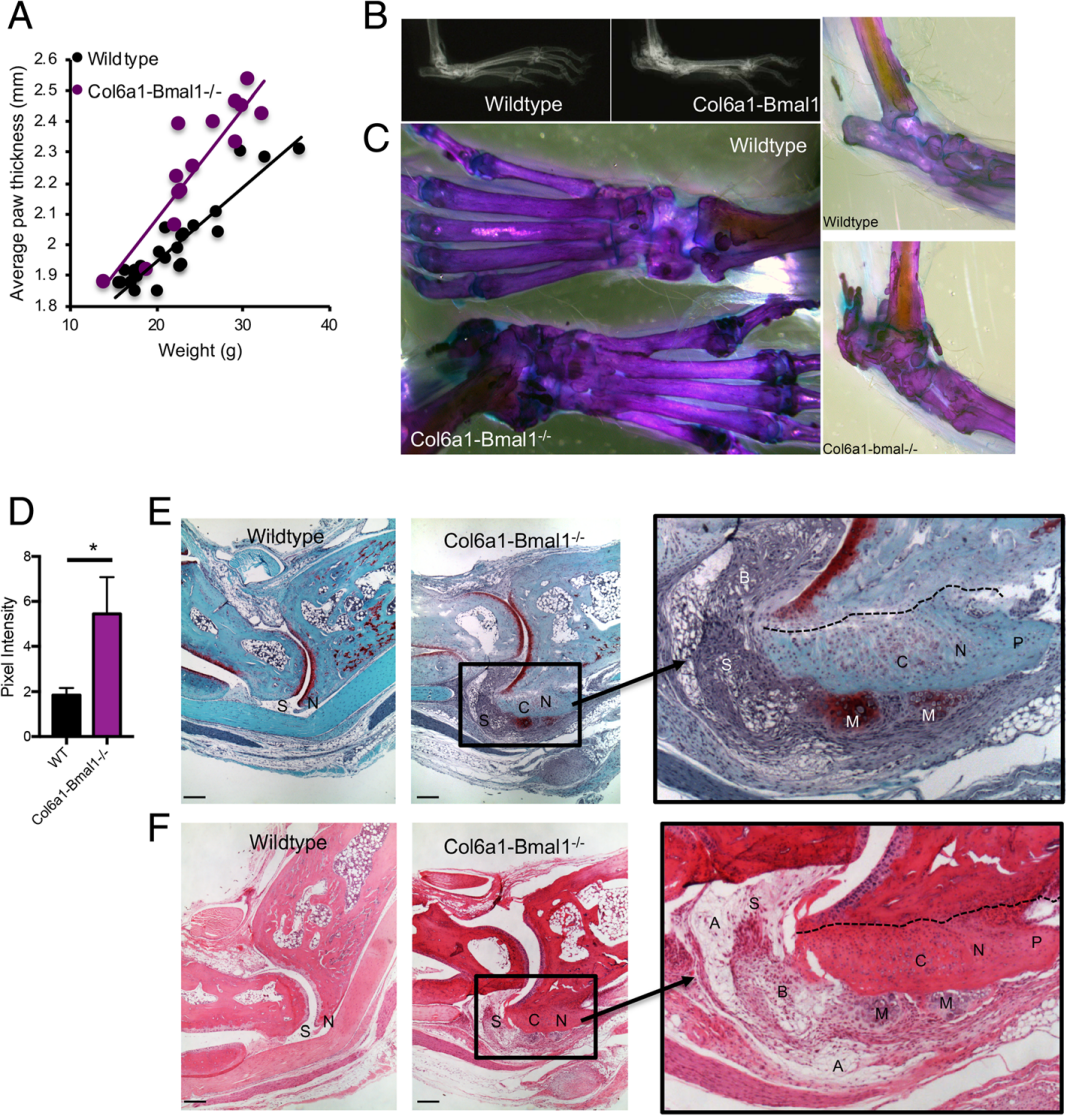
Effect of *Bmal1* deletion in joint mesenchymal cells on joint development. **a** Hind paw thickness (in mm) plotted against animal weight (in g) measured in male (7 wild-type, 8 Col6a1-Bmal1^−/−^) and female (18 wild-type, 7 Col6a1-Bmal1^−/−^) animals. **b** X-ray images at 9 months. **c** Whole-mount staining of the hind limbs from 9-month-old wild-type and Col6a1-*Bmal1*^−/−^ mice. **d** Quantification of pixel intensity from x-rays of 9-month-old mice in an ROI superior to the calcaneus (*n* = 4–6 limbs). Analysis was by unpaired *t* test. **e** Safranin O staining of ankle joints from 14- to 17-week-old mice where cartilage is stained red. Scale bar represents 500 μm. **f** H&E staining of ankle joints from 14- to 17 week old mice. Scale bar represents 500 μm. Col6a1-Bmal1^−/−^ mice show thickened synovium (S); the enthesis (N) is eroded (*dashed line* delimits the interface between eroded bone and repair tissue), and the erosion is filled with exuberant chondro-osseous repair tissue (C) with the formation of a spur (P); there is chondroid metaplasia (M) at the junction of the synovium, joint capsule and enthesis repair tissue. The chondroid metaplasia differs from the chondro-osseous repair tissue in its morphology and tinctorial reaction with Safranin O. The synovium is fibrotic (B) and contains a mixture of spindle and round connective tissue cells. At its interfaces with connective tissues other than metaplastic chondroid tissue, there is a marked increase in adipocytes (A)

Col6a1-Bmal1^−/−^ animals showed thickening of the hind footpad (Fig. 3a), and this was evident in both sexes (Additional file 1: Figure S2a). X-ray imaging (Fig. 3b and Additional file 1: Figure S2b) and whole-mount staining (Fig. 3c) revealed density changes around the tarsocrural joint. There was also evidence of calcification of intervertebral disk space within the spine and tail (Additional file 1: Figure S2c and d), in keeping with observations in global *Bmal1*^-/-^ animals [5] and mice lacking *Bmal1* in cells of the intervertebral disk (including chondrocytes and cells of the annulus fibrosus, which are morphologically similar to fibroblasts) [24, 25]. By 9 months, there were signs that the phenotype could affect mobility and general well-being; consequently mice were not maintained beyond this age. Quantitative analysis of X-ray images of the hind limbs confirmed increased bone density in a defined region above the calcaneus in Col6a1-Bmal1^−/−^ hind limbs (Fig. 3d). There was an increase in calcified tissue in the joint spaces between the metatarsal and phalangeal bones and within the ankles. A calcaneal spur was evident from 6 months. Similar observations had been reported in global *Bmal1*^−/−^ mice, but the cell type responsible had not been determined [5]. In keeping with this, we observed that global *Bmal1* deletion [19] also resulted in increased paw thickness, abnormalities between the metatarsal and phalanges, and a calcaneal spur (Additional file 1: Figure S3). Histological assessment of Col6a1-Bmal1^−/−^ ankle joints in 14- to 17-week-old mice (Fig. 3e, f) revealed three distinctive changes restricted to the tarsocrural joint: (1) a thickening of the synovial subintima, in the absence of an increase in the number of surface synoviocytes; (2) erosion and exuberant chondro-osseous repair of the entheses at the condyles of the distal tibia with the formation of a spur; and (3) nodular chondroid metaplasia at the interface between the synovium, the ankle joint capsule and the damaged/healing entheses. Morphologically, there was minimal neutrophilic or lymphocytic inflammation either in the thickened synovium or at the site of the enthesopathy. The thickened synovium was generally fibrotic, but at its junction with bone and capsule consisted of adipose tissue. It contained a mixture of round and spindle-shaped connective tissue cells and infrequent cells resembling histiocytes. The disordered cartilage was at various stages of maturation, with many cells large and hypertrophic (a phenotype associated with endochondral ossification). These changes reflect observations by Bunger et al. in the knees of global *Bmal1*^*−/−*^ mice, describing areas of calcification and ossification around tendons and ligaments closely associated with bone insertion sites [5].

**Fig. 4:**
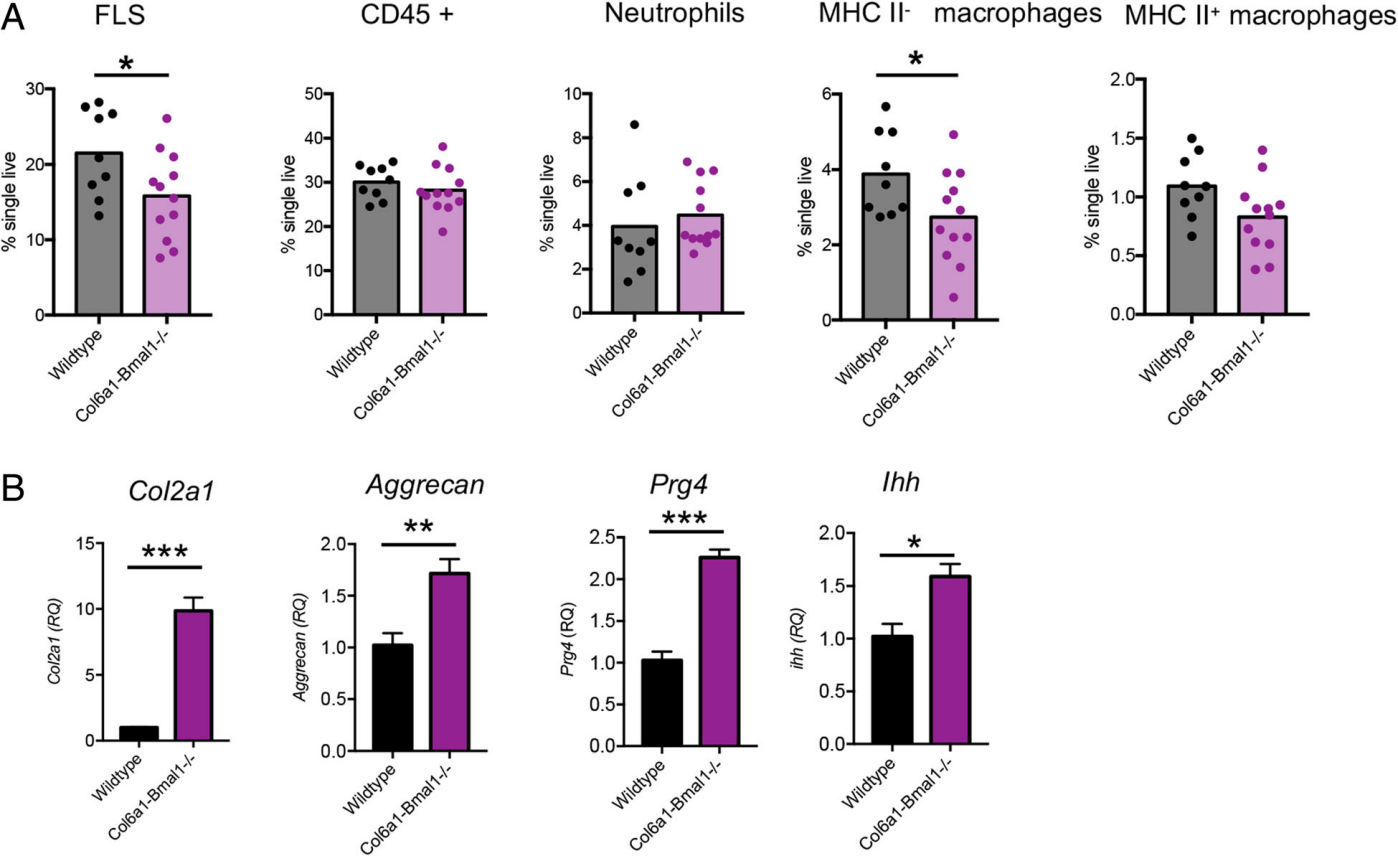
*Bmal1* deletion alters the balance of fibroblasts and chondrocytes within joints. **a** Flow cytometric analysis of the cellular composition of the joints from wild-type (*n* = 9) and Col6a1-*Bmal1*^−/−^ (*n* = 12) mice (aged 9–18 weeks) after exclusion of doublets and dead cells. Analysis by unpaired *t* test. **b** Expression of chondrocyte-associated genes in the hind limbs of mice, aged 10 weeks (*n* = 4). Analysis by unpaired *t* test

To further investigate these histological changes, cells were collagenase-digested from limbs for flow cytometric analysis. Strikingly, numbers of FLS were markedly reduced in Col6a1-Bmal1^−/−^ joints, supporting histological evidence for a phenotypic switch from FLS to chondrocytes (Fig. 4a). Neutrophil numbers were consistent between genotypes. In keeping with others [26] we identified two populations of macrophages within the joints based on differential expression of major histocompatibility complex (MHC) class II. Numbers of MHC II^−^ macrophages, which make up the majority of the macrophages found within the synovial lining and are considered true tissue-resident macrophages, were reduced in Col6a1-Bmal1^−/−^ mice. Numbers of MHC II^+^ macrophages, considered to originate in the bone marrow, although reduced, were not significantly altered in Col6a1-Bmal1^−/−^ animals. qPCR analysis of whole hind limbs revealed that chondrocyte-associated genes were significantly upregulated in Col6a1-Bmal1^−/−^ animals. Expression of *Col2a1*, *aggrecan*, *proteoglycan 4* and *Indian hedgehog* were significantly increased within the hind paws in the absence of mesenchymal cell *Bmal1*, further indicating increased numbers of active chondrocytes (Fig. 4b).

### Bmal1 in FLS represses inflammatory arthritis

**Fig. 5:**
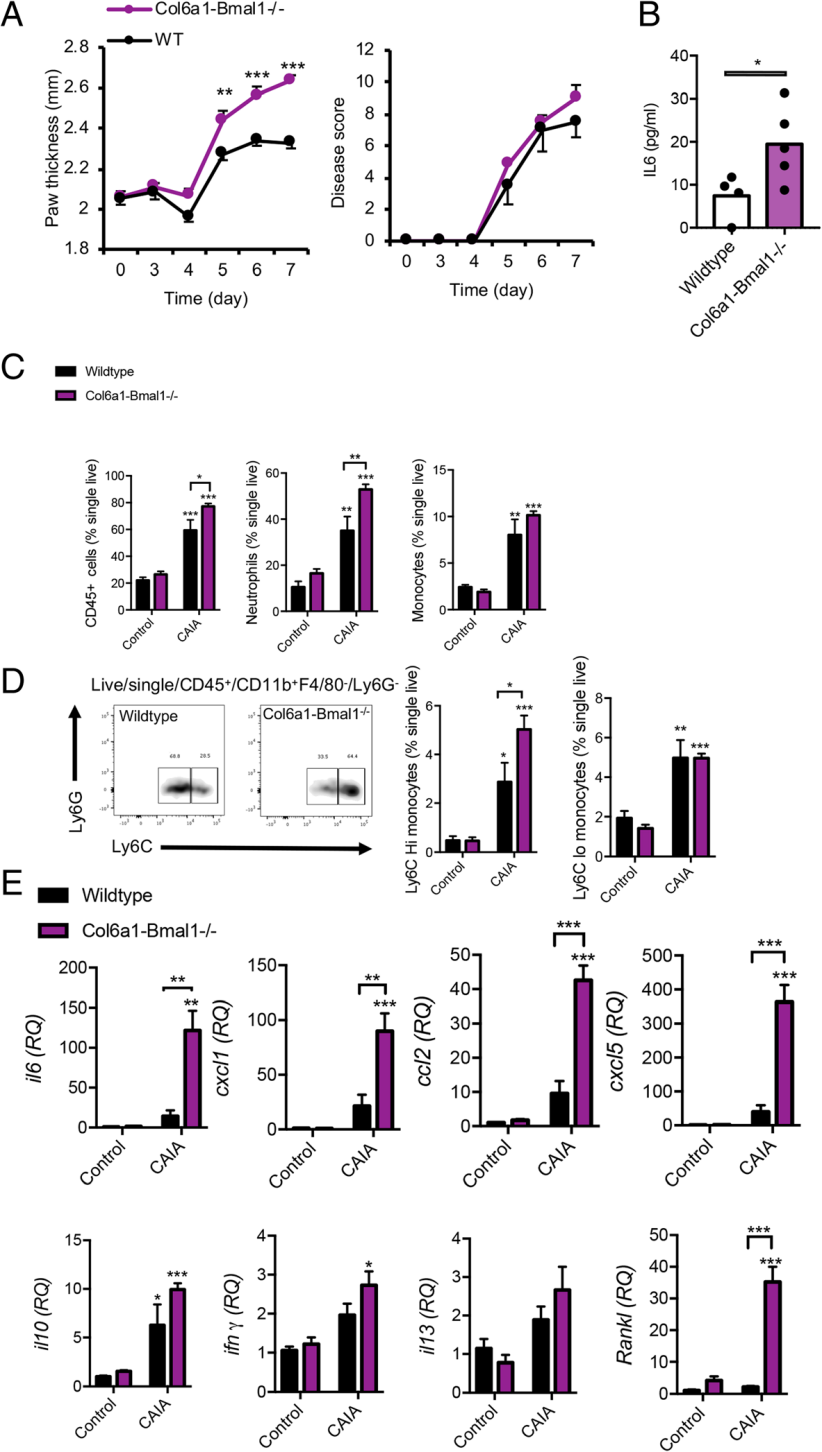
Enhanced response of Col6a1-Bma1^−/−^ mice to collagen antibody-induced arthritis. **a** Hind paw thickness and disease score after initiation of collagen antibody-induced arthritis (CAIA) in wild-type (n = 4) and Col6a1-Bmal1^−/−^ (*n* = 5) mice. One-way analysis of variance (ANOVA) and Bonferroni multiple comparisons. **b** Serum IL-6 levels in CAIA animals at the time they were killed on day 7. Analysis by *t* test. **c** Quantification of leukocytes within hind paws of arthritic (*n* = 4–5/group) and non-arthritic (*n* = 4/group) animals. Two-way ANOVA and post hoc Bonferroni correction. Stars directly above CAIA bars indicate significant difference compared with control counterparts. **d** Representative plots showing increased presence of LycC^hi^ monocytes in inflamed limbs of Col6a1-Bmal1^−/−^ mice, and quantification of Ly6C^hi^ and Ly6C^lo^ monocytes within hind paws of arthritic (*n* = 4–5/group) and non-arthritic (*n* = 4/group) animals. Two-way ANOVA and post hoc Bonferroni correction. Stars directly above CAIA bars indicate significant differences compared with control counterparts. **e** Transcripts of inflammatory cytokines in hind limbs harvested from control (*n* = 4/group) and CAIA mice (*n* = 4–5/group). Two-way ANOVA and post hoc Bonferroni correction. Stars directly above CAIA bars indicate significant difference compared with control counterparts

To investigate the role of *Bmal1* in joint mesenchymal cells under inflammatory conditions, arthritis was induced using a cocktail of collagen antibodies. Disease was evident 5 days after administration. There was no genotypic difference in disease score; however, swelling of affected paws was greater in Col6a1-Bmal1^−/−^ animals than in wild-type animals (Fig. 5a). Serum IL-6 concentrations were also significantly higher in Col6a1-Bmal1^−/−^ animals (Fig. 5b), in keeping with a more aggressive inflammatory arthritis. Infiltrating neutrophils and Ly6C^hi^ (but not Ly6C^lo^) monocytes were increased in arthritic Col6a1-Bmal1^−/−^ limbs (Fig. 5c and d). In keeping with this, pro-inflammatory cytokine transcripts (*Il6*, *Cxcl1*, *Ccl2*, *Cxcl5*) and *receptor activator of nuclear factor κ ligand* (*Rankl*) transcript levels were significantly higher. There were no discernible genotypic differences in cytokines that have been attributed to an anti-inflammatory role in inflammatory arthritis (*Il10*, *Ifnγ* and *Il13*) [27–29] (Fig. 5e).

**Fig. 6:**
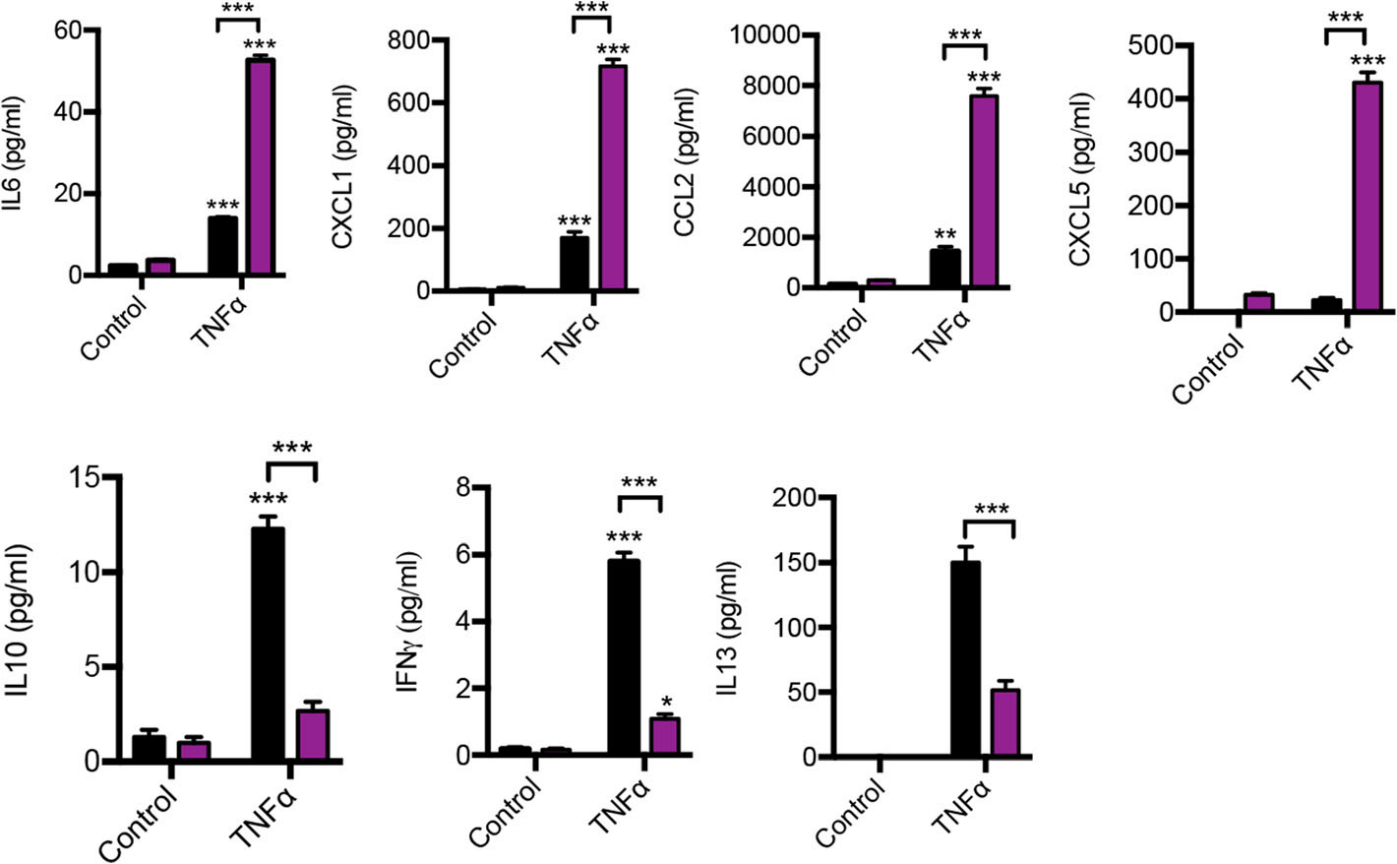
Altered response of fibroblast-like synoviocytes (FLS) lacking *Bma1*^−/−^ to inflammatory stimuli. FLS were cultured from Col6a1-*Bmal1*^−/−^ and wild-type littermates to passage 3 and purified for CD90.2 expression before stimulation for 8 h with tumour necrosis factor (TNF)-α. Expression of inflammatory cytokines in cell supernatants was assessed by Bioplex and enzyme-linked immunosorbent assay (CXCL5 only) (*n* = 5–6). One-way analysis of variance and post hoc Bonferroni correction. Stars directly above TNF-α bars indicate significant difference compared with control counterparts

To assess how FLS contribute to this enhanced inflammatory response, cultured, purified FLS were stimulated with the pro-inflammatory cytokine TNF-α, and the supernatant was analysed for cytokines by performing a Bio-Plex assay (Fig. 6 and Additional file 1: Table S4). After stimulation, *Bmal1*^−/−^ FLS secreted higher levels of IL-6, CXCL1, CCL2 and CXCL5, and levels of cytokines with known anti-inflammatory properties in inflammatory arthritis (IL-10, interferon [IFN]-γ and IL-13) were dampened. Interestingly, under basal conditions, *Bmal1* loss results in increased secretion of several pro-inflammatory cytokines (e.g., CCL2 and CXCL5), but this did not reach statistical significance. Together these data suggest disruption of the basal inflammatory state in FLS in the absence of *Bmal1*, as well as an enhanced pro-inflammatory response in a stimulatory environment.

## Discussion

This study addresses the role of the core clock component *Bmal1* in defining musculoskeletal structure. By deleting *Bmal1* in a targeted cell population, we were able to identify the cell lineage responsible for a previously recognised joint arthropathy. *Bmal1* deletion in joint mesenchymal cells rendered FLS and articular cartilage arrhythmic and led to disruption of ankle joint architecture. This was evidenced by focal chondroid metaplasia, decreased resident synovial macrophage numbers, and altered basal secretion of a subset of cytokines by FLS in the native state.

Global *Bmal1*-knockout mice show a progressive ectopic mineralisation with new bone formation at tendon and ligament insertion sites [5]. Our findings suggest this ossification may be a consequence of a change in mesenchymal stem cell differentiation towards a bone-depositing chondroid phenotype. These pathological chondrocytes drive calcification of the tendon sheath to form a calcaneal spur. Global deletion of *Bmal1* in adult mice does not result in this abnormal calcification [7]; therefore, it is likely a consequence of the absence of *Bmal1* in mesenchymal cells during embryogenesis/early post-natal development. Articular chondrocytes and synovial fibroblasts are derived from common joint progenitor cells, with the action of different factors driving them towards a chondrogenic or anti-chondrogenic lineage [30]. Furthermore, Col6a1 is present in synovial membrane and articular cartilage in 16.5-day murine embryos [31]. We suggest that BMAL1 expression in joint mesenchymal cells may be essential for regulating the balance between FLS and chondrocytes of the developing embryo. In support of this, higher expression levels of chondrocyte-associated genes were detected in the hind limbs of Col6a1-Bmal1^−/−^ mice. This includes genes encoding for type II collagen and aggrecan (major structural components of cartilage), proteoglycan 4 (synthesised by chondrocytes) and Indian hedgehog (associated with chondrocyte differentiation, proliferation and maturation). Intriguingly, others have shown that targeted ablation of *Bmal1* in mouse chondrocytes causes progressive degeneration of articular cartilage within the knee, with loss of chondrocytes and extracellular matrix evident from 3 months of age [32]. Taken together, it is clear that *Bmal1* within mesenchymal cells is critical for joint health throughout development and later life.

Col6a1-Bmal1^−/−^ animals demonstrated further alterations in the cellular composition of the synovial tissue. Tissue-resident MHC II^−^ macrophages were significantly reduced within the joints. Resident synovial macrophages play critical roles in immune surveillance, maintenance of tissue integrity and limiting inflammation. It should be noted that human and murine macrophages express *Col6a1* and secrete collagen VI protein [33, 34]. Macrophages derived from Col6a1-Bmal1^−/−^ mice express low levels of *Col6a1* and *Cre* (data not shown). However, Cre expression was insufficient to drive recombination and did not affect expression of the targeted exon of *Bmal1 (*exon 8) within the macrophage (Additional file 1: Figure S6a). In keeping with this, expression of BMAL1 protein was unchanged (Additional file 1: Figure S6b). Furthermore, Col6a1-Bmal1^−/−^ macrophages showed robust circadian rhythms in PER2::luc bioluminescence (Additional file 1: Figure S6c). (Previous work demonstrated that efficient deletion of *Bmal1* renders these cells arrhythmic [18].) Thus we suggest that the observed change in numbers of MHC II^−^ macrophages within the joints is a consequence of loss of BMAL1 in the mesenchymal cell population. The differentiation and function of resident synovial macrophages is governed by (as yet undetermined) tissue-specific cues most likely derived from FLS [35]. Our data suggest that *Bmal1* deletion in mesenchymal joint cells indirectly affects the joint macrophage population by disrupting signalling between FLS and synovial macrophages. Indeed, we saw that in stimulated FLS the expression of granulocyte-macrophage colony-stimulating factor, which regulates the proliferation and differentiation of macrophages, was significantly decreased in the absence of *Bmal1* (Additional file 1: Table S4). Conversely, M-CSF levels released from FLS lacking *Bmal1* were heightened. Taken together, this suggests that *Bmal1* is important for the regulation of cytokine signals from FLS which facilitate the proliferation, differentiation and survival of macrophages within the synovium. The disordered macrophage population may contribute to the destructive joint phenotype, and may also affect the propagation and resolution of inflammatory arthritis.

FLS are key players in inflammatory arthritis, releasing a range of pro-inflammatory mediators. Chondrocytes respond to these mediators (e.g., TNF-α and IL-1) by releasing matrix-degrading proteinases, resulting in joint damage. Chondrocytes do also have the capacity to release pro-inflammatory cytokines in response to stimulation [36], and so may also contribute as a cellular source of inflammatory mediators which damage the joint [37]. Col6a1-Bmal1^−/−^ mice showed enhanced localised inflammation after induction of arthritis. In comparison to wild-type counterparts, the affected limbs exhibited enhanced swelling, increased expression of pro-inflammatory cytokines (e.g., *Ccl2*, *Cxcl1* and *Cxcl5*) and increased infiltration of neutrophils and Ly6C^hi^ monocytes. Both Ly6C^hi^ and Ly6C^lo^ monocytes were recruited to the joints during collagen antibody-induced arthritis. Interestingly, we did not see significant differences in numbers of infiltrating Ly6C^lo^ monocytes between the two genotypes, only Ly6C^hi^. Recent work has established that Ly6C^lo^ monocytes have an increased potential to differentiate into osteoclasts and inflammatory macrophages, which play a key role in the destruction of articular bone during arthritis [26, 38]. Furthermore, given the increased levels of *Rankl* (which plays a role in bone erosion) in the arthritic joints of Col6a1-Bmal1^−/−^ animals, it would be interesting to determine, in future studies, the effects of loss of *Bmal1* in joint mesenchymal cells on bone erosion. CCL2 drives monocyte recruitment, whilst CXCL1 and CXCL5 are neutrophil chemoattractants and thus likely explain the observed increase in monocytes and neutrophils. This more severe inflammatory phenotype extended to a systemic response, because arthritic Col6a1-Bmal1^−/−^ mice exhibited increased levels of circulating IL-6. Some stromal cells within secondary lymphoid organs (Peyer’s patches and isolated lymphoid follicles) express Col6a1 [39]. In control experiments, qPCR analysis confirmed the expression of *Col6a1* in Peyer’s patches of wild-type and Col6a1-Bmal1^−/−^ animals (Additional file 1: Figure S1b) and *Cre* expression in Col6a1-Bmal1^−/−^ animals only (data not shown). However, this did not affect expression of *Bmal1* (Additional file 1: Figure S1b). Consequently, this increase in systemic IL-6 is most likely a consequence of targeting of joint-resident stroma rather than an extra-articular source.

In vitro studies demonstrated that loss of *Bmal1* in FLS promotes enhanced secretion of pro-inflammatory cytokines and reduced secretion of anti-inflammatory cytokines after stimulation, suggesting that *Bmal1* within FLS is critical for restraining joint inflammation. Cultured Col6a1-Bmal1^−/−^ FLS demonstrated elevated release of a subset of pro-inflammatory cytokines under naïve conditions. This reflects observations in pulmonary Club cells [40], macrophages and monocytes [41], where BMAL1 regulates recruitment of the glucocorticoid receptor (Club cells) and the polycomb repressive complex (macrophages/monocytes) to target genes, resulting in repression of *Cxcl5* and *Ccl2* transcription, respectively. Given the role of *Bmal1* in restraining joint inflammation, it is important to consider the effects of the chronic inflammatory environment on *Bmal1* expression. It is reported that TNF-α induces the expression of *Bmal1* in human synovial fibroblasts via calcium-dependent pathways [11, 15]. Similarly, studies have shown higher levels of BMAL1 protein in synovium from patients with rheumatoid arthritis than in synovium from patients with osteoarthritis [13]. In light of our findings, we suggest that this enhanced BMAL1 expression in rheumatoid arthritis may in fact act to dampen inflammation.

## Conclusions

In the present study, we demonstrated the critical importance of *Bmal1* in joint mesenchymal cells in regulating FLS and chondrocyte development within the joints. Our data provide a mechanism to explain the abnormal bone phenotype and altered cellular composition of the joint associated with pre-natal deletion of *Bmal1.* Additionally, we identified a role for *Bmal1* in FLS for restraining local responses to inflammation and highlighted a role for the circadian clock in regulating inflammatory arthritis.

## Additional file

Additional file 1: Supplementary data. (PDF 2885 kb)

## Abbreviations

Cry: Cryptochrome
ELISA: Enzyme-linked immunosorbent assay
FLS: Fibroblast-like synoviocytes
IFN: Interferon
IL: Interleukin
M-CSF: Macrophage colony-stimulating factor
MHC: Major histocompatibility complex
PE: Phycoerythrin
Per: Period
RANKL: Receptor activator of nuclear factor κ ligand
TNF-α: Tumour necrosis factor-α
ZT: Zeitgeber time
