# Supplementary material for: The circadian regulator Bmal1 in joint mesenchymal cells regulates both joint development and inflammatory arthritis
Source: Arthritis Res Ther. 2019 Jan 6;21:5. doi: 10.1186/s13075-018-1770-1 (PMC6322248; doi:10.1186/s13075-018-1770-1)
Supplement: Supplementary file 1 — Supplementary data. (PDF 2885 kb) [file 13075_2018_1770_MOESM1_ESM.pdf]

Supplementary Figure 1: (A) Bioluminescene recording from lung fibroblasts cultured from Col6a1-Bmal1$^{-/-}$ and wildtype animals on a PER2::luc background. (B) QPCR analysis of *Col6a1* and *Bmal1* expression in Peyer's patches isolated from wildtype (n=5) and Col6a1-Bmal1$^{-/-}$ (n=3) animals.

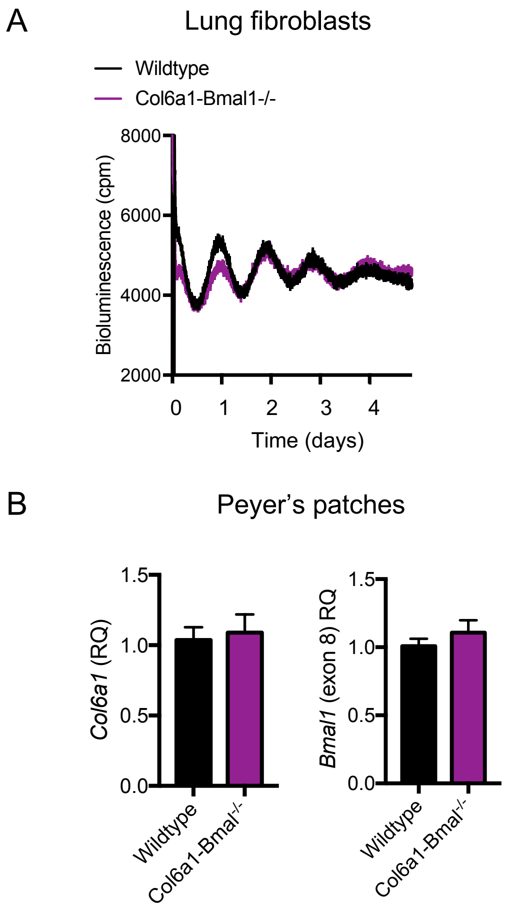

Supplementary Figure 2: (A) Hind paw thickness (mm) plotted against animal weight (g) measured in female (top) and male (bottom) Col6a1-Bmal1$^{-/-}$ and WT mice (pooled data shown in Figure 2a). (B) X-rays of hind limbs from Col6a1-Bmal1$^{-/-}$ mice and WT littermates age 3 and 6 months, representative of n=3 animals/genotype. Arrow indicates location of a calcaneal spur. (C) X-ray images of the tails of Col6a1-Bmal1$^{-/-}$ mice and WT littermates age 3, 6 and 9 months, arrows indicate areas of calcification within the intervertebral disks. (D) Pixel intensity in a defined region of interest encompassing the intervertebral disk space was quantified in 9 month old mice, n=3/genotype.

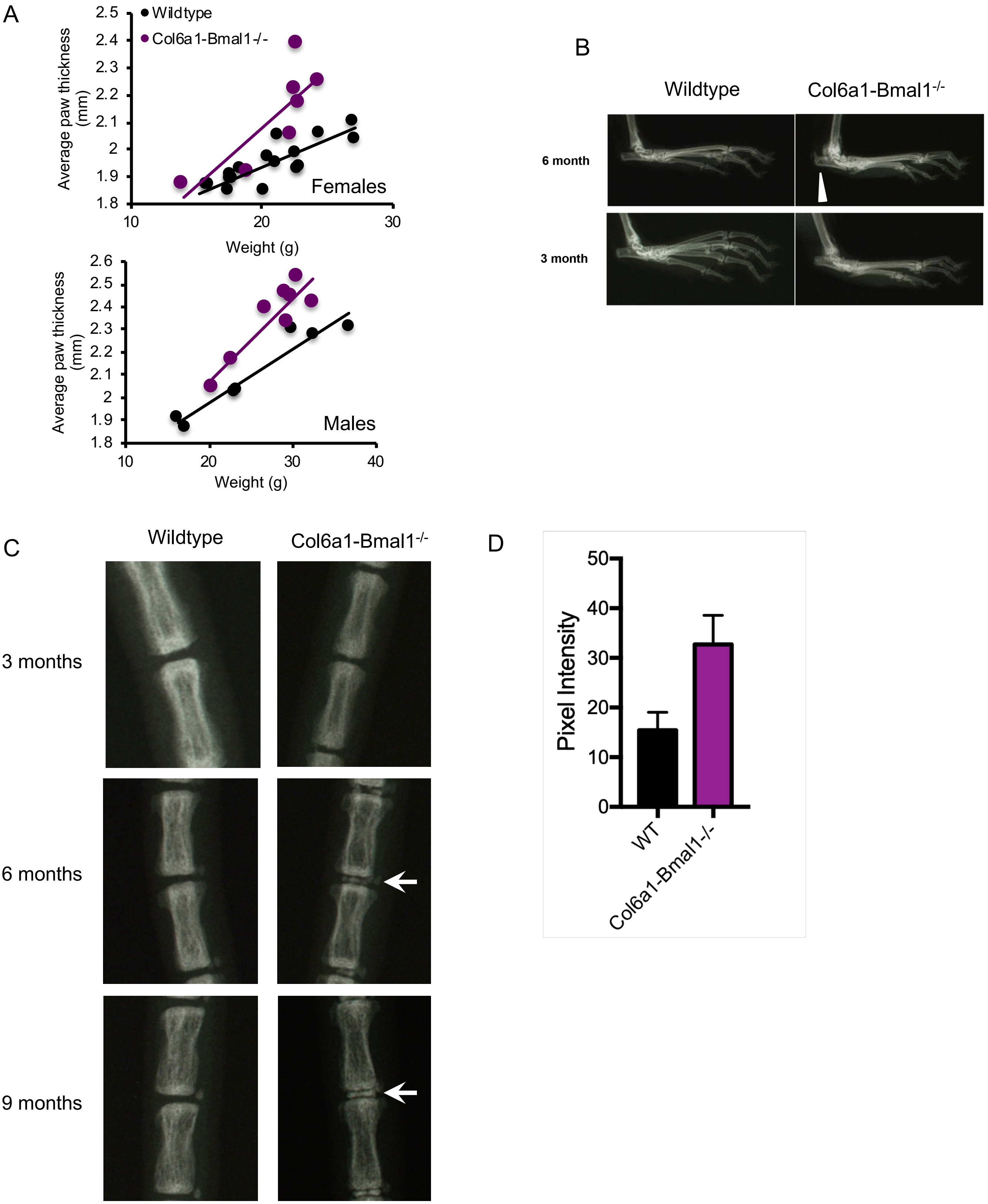

Supplementary Figure 3: (A) Comparison of paw thickness in male wildtype (Bmal1$^{+/+}$, n=9) and KO (*Bmal1*$^{-/-}$, n=5) mice. Paw thickness (mm) and weights (g) are plotted against one another. (B) X-rays of hind limbs from male *Bmal1*$^{-/-}$ and wildtype littermates, age 4-5 months. A spur protruding from the calcaneum of the *Bmal1*$^{-/-}$ is marked by an arrow, representative of n=3-4 animals/genotype. (C) Quantification of pixel intensity from X-ray images in a region of interest superior to the calcaneus, n=6-8 limbs, unpaired T test.

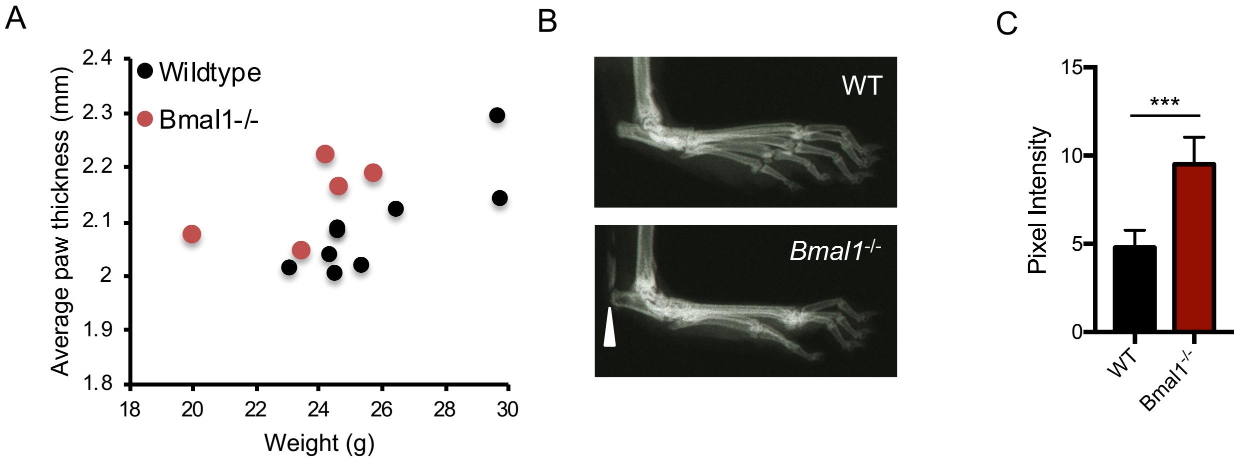

Supplementary Figure 4: Gating strategy for identification of cells digested from the hind limbs of naïve animals. Single cells were gated as shown below to identify populations of FLS (CD45^{-}CD90.2^{+}), T cells (CD45^{+}CDllb^{-}CD3^{+}), neutrophils (CD45^{+}CDllb^{+}Ly6C^{+}Ly6G^{+}) and MHCII^{high} and MHCII^{low} macrophages (CD45^{+}CD11b^{+}Ly6C^{+}CD64^{+}F4/80^{+})

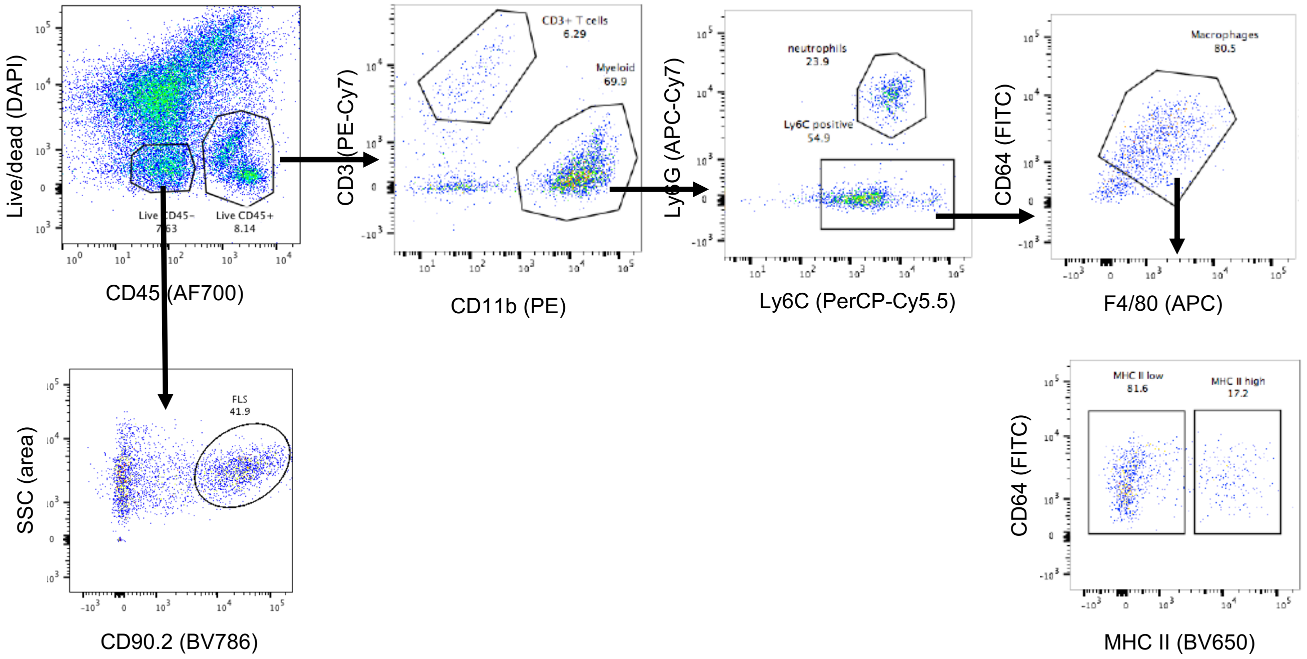

Supplementary Figure 5: Gating strategy for identification of cells digested from the hind limbs of arthritic animals. Single cells were gated as shown below to identify populations of neutrophils (CD45^{+}CDllb^{+}F4/80^{-}Ly6C^{+}Ly6G^{+}) and Ly6C^{hi} and Ly6C^{low} monocytes (CD45^{+}CD11b^{+} F4/80^{-}Ly6G^{-}).

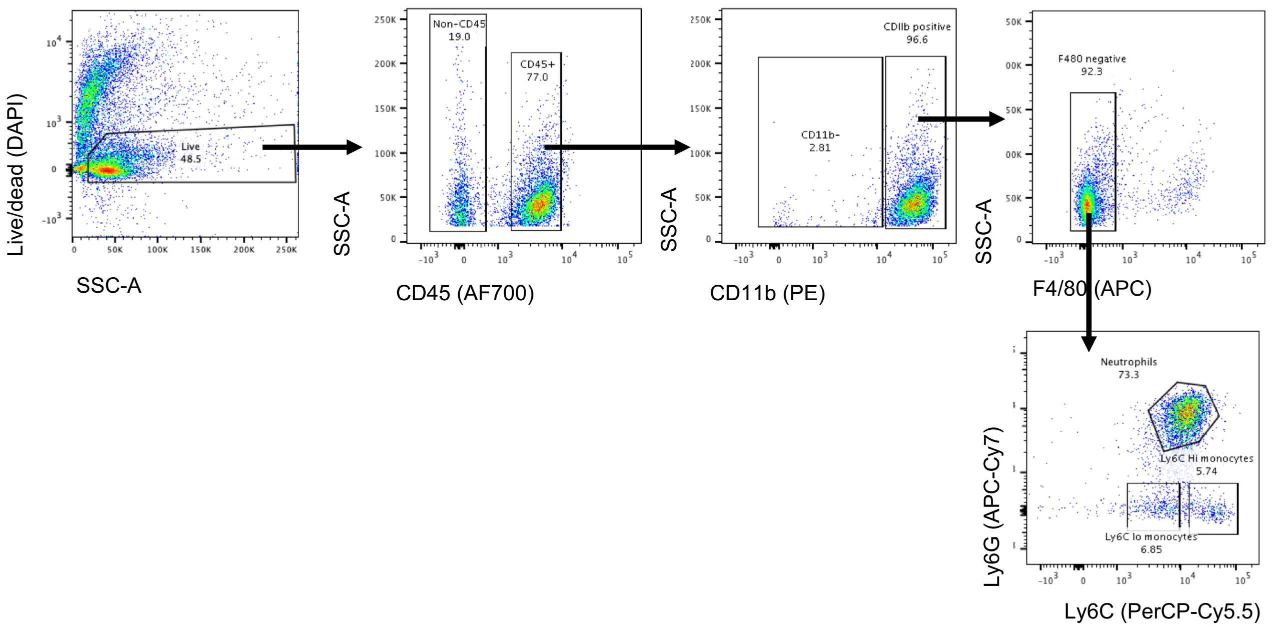

Supplementary Figure 6: Expression of BMAL1 is unaffected in macrophages**.** (A) Expression of *Bmal1* (exon 8) was quantified from peritoneal macrophages harvested from Col6a1-Bmal1$^{-/-}$ (3 male and 3 female) and wildtype (4 male and 3 female) mice using QPCR, *gapdh* was utilized as a housekeeping gene and levels were normalized to wildtype expression. (B) Western blot of BMAL1 and β-ACTIN protein levels in bone marrow derived macrophages harvested from Col6a1-Bmal1$^{-/-}$ and wildtype animals. (C) Bioluminescence analysis of PER2::luciferase activity in peritoneal macrophages from Col6a1-Bmal1$^{-/-}$ mice and wildtype counterparts, representative of n=3-4 males/genotype.

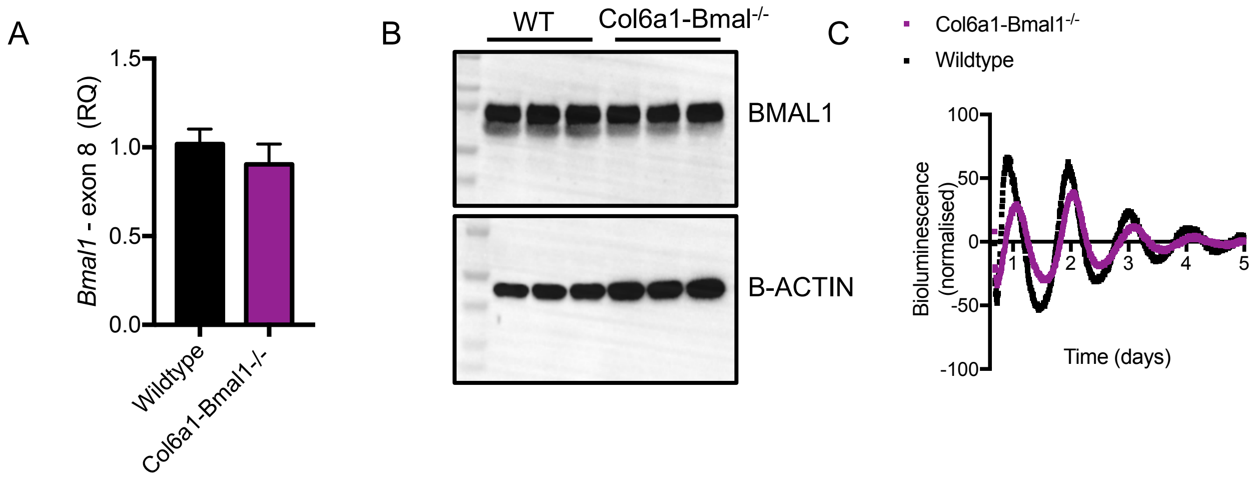

Supplementary Table 1: Antibodies utilized for flow cytometric assessment of cells digested from naïve limbs

Antibody	Fluorophore	Clone	Supplier
CD45	AF700	30-F11	BD Biosciences
CD3	Pe-Cy7	145-2C11	Biolegend
CD11b	PE	M1/70	Biolegend
Ly6G	APC-Cy7	IA8	BD Biosciences
Ly6C	PerCP-Cy5.5	AL-21	Biolegend
CD64	FITC	XS4-5/7.1	Biolegend
F4/80	APC	BM8	eBioscience
MHC II	BV650	M5/114.15.2	BD Biosciences
CD90.2	BV786	30-H12	Biolegend

Supplementary Table 2: Antibodies utilized for flow cytometric assessment of cells digested from arthritic limbs.

Antibody	Fluorophore	Clone	Supplier
CD45	AF700	30-F11	BD Biosciences
CD11b	PE	M1/70	Biolegend
F4/80	APC	BM8	eBiosciences
Ly6G	APC-Cy7	IA8	Biolegend
Ly6C	PerCP-Cy5.5	AL-21	BD Biosciences

Supplementary Table 3: Primers utilized for QPCR analysis. *Indicates where TaqMan gene expression assays (ThermoFisher Scientific) were utilized with the relevant product code.

	Forward	Reverse	Probe (Fam-Tamra)
Gapdh	CAATGTGTCCGTCGTCGATCT	GTCCTCAGTGTAGCCCAAGATG	CGTGCCGCCTGGAGAAACCTG CC
Per2	GCCTTCAGACTCATGATGACA GA	TTTGTGTGCGTCAGCTTTGG	ACTGCTCACTACTGCAGCCGCTC GT
Bmal1 (exon 8)	CGTCGGGACAAAATGAACAG	GAACAGCCATCCTTAGCAC	TACCAACATGCAATGCAATGT CCAGGAA
Cry1	CTGGCGTGGAAGTCATCGT	CTGTCCGCCATTGAGTTCTATG	CGCATTTCACATACACTGTAT GACCTGGACA
Dbp	CCGTGGAGGTGCTAATGACCT	CCTCTGAGAAGCGGTGCCT	TGAACCTGATCCGGCTGATCTTG CC
Il6	CTATACCACTTCACAAGTCGGAGG	TGCACAACTCTTTTCTCATTTCC	TTAATTACACATGTTCTCTGGGAA ATCG
Cxcl1	CTGCACCCAAACCGAAGTC	AGCTTCAGGGTCAAGGCAAG	CACTCAAGAATGGTCGCGAGGC
Ccl2	TTCTGGGCCTGCTGTTCA	CCAGCCTACTCATTGGGATCA	CTCAGCCAGATGCAGTTAACG CCCC
Ifn gamma	TCAAGTGGCATAGATGTGGAAGAA	TGGCTCTGCAGGATTTTCATG	TCACCATCCTTTTGCCAGTTC CTCCAG
Rankl	GCACACCTCACCATCAATGCT	GGTACCAAGAGGACAGAGTGACTTT A	CCAGCATCCCATCGGGTTCCC
Rev-erb α*	Mm00520708_m1		
Col2a1 *	Mm01309565_m1		
Col6a1*	Mm00487160_m1		
Aggrecan *	Mm00545794_m1		
Prg4 *	Mm01284582_m1		
Ihh *	Mm00439613_m1		
Cxcl5 *	Mm00436451_g1		
Il10 *	Mm01288386_m1		
Il13 *	Mm00434204_m1		

Supplementary Table 4: Cytokine secretion by cultured FLS. Cell supernatants were assayed by Bioplex or ELISA (CXCL5 and M-CSF), n=4-6. Data was analyzed using two way ANOVA and post hoc Bonferroni's test (black asterix) or unpaired T test (grey asterix). Effects of genotype are shown within each treatment group (control and + TNFα). Secretion of IL1α, IL1β, IL2, IL3, IL4, IL5, IL12p40, IL17, CCL11, G-CSF and CCL3 were minimal and thus data are not shown.

	**Control**		**+ TNFα**	
	**WT (pg/ml)**	**Col6a1-Bmal1$^{-/-}$ (pg/ml)**	**WT (pg/ml)**	**Col6a1-Bmal1$^{-/-}$ (pg/ml)**
**IL6**	2.3 ± 0.1	3.7 ± 0.2	13.9 ± 0.5	52.6±1.2***
**IL9**	3.5 ± 0.9	2.4 ± 0.4	11.2 ± 0.8	6.4±0.9**
**IL10**	1.3 ± 0.4	1.0 ± 0.3	12.3 ± 0.6	2.7±0.5***
**IL12 p70**	5.8 ± 1.6	5.5 ± 1.3	33.3 ± 2.3	24.1±0.8**
**IL13**	ND	ND	150.4 ± 12.1	51.7 ± 7.2***
**GM-CSF**	ND	ND	15.1 ± 0.8	11.2 ± 0.7**
**IFNɣ**	0.2 ± 0.06	0.2 ± 0.05	5.8 ± 0.3	1.1 ± 0.1***
**CXCL1**	6.7 ± 0.7	11.7 ± 0.4	170.7 ± 19.0	716.7 ± 22.1***
**CCL2**	138.0 ± 6.6	303.7 ± 14.5	1447.7 ± 194.4	7586.9 ± 298.4***
**CCL4**	1.3 ± 0.072	3.9 ± 0.3***	5.2 ± 0.3	11.1 ± 0.3***
**CCL5**	29.4 ± 2.3	29.6 ± 3.3	387.6 ± 39.1	1574.5 ± 54.7***
**TNFα**	7.2 ± 0.3	4.9 ± 0.2	13777.5 ± 587.2	14762.7 ± 449.9
**CXCL5**	1.1 ± 0.2	32.9 ± 2.8	22.6 ± 4.3	430.6 ± 19.1***
**M-CSF**	ND	15.9 ± 3.0	2.4 ± 1.0	18.5 ± 6.7*
